# Cryptocurrency and stock market: bibliometric and content analysis

**DOI:** 10.1016/j.heliyon.2022.e10514

**Published:** 2022-09-02

**Authors:** Saeed Sazzad Jeris, A.S.M. Nayeem Ur Rahman Chowdhury, Mst. Taskia Akter, Shahriar Frances, Monish Harendra Roy

**Affiliations:** Department of Business Administration, Shahjalal University of Science & Technology, Sylhet, Bangladesh

**Keywords:** Cryptocurrency, Stock market, VOSViewer, Bibliometric analysis, Content analysis, Systematic review

## Abstract

This study conducted a systematic review regarding the association between cryptocurrency and the stock market. This study used bibliometric and content analysis covering 151 articles from 2008 to November 2021. Using VOSviewer software, we explored the influential aspects of the literature, such as the prominent institutions, authors, countries, and journals. Additionally, we performed co-authorship, bibliographic coupling, and co-occurrence of keywords to understand the network. Furthermore, in the content analysis, we discussed key findings of four major research streams that we identified. Finally, we present seven research questions that can be explored in the future. The findings have a number of implications for the present state of the literature on cryptocurrency and the stock market, including study gaps and potential future research initiatives.

## 1 Introduction

Cryptocurrencies have become a worldwide phenomenon constantly discussed in the media, venture capitalists, banking, stock market, political organizations, etc (Glaser et al., 2014). Cryptocurrencies have recently arisen new financial asset class, and this provides a chance to research uncovered features of cryptocurrencies.

**Figure 1:**
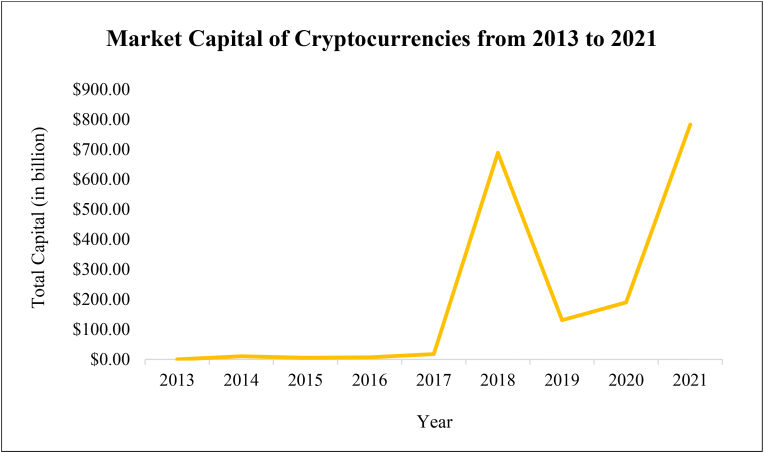
Market capital of cryptocurrencies.

Virtual currency like cryptocurrency has carved itself a distinct position in the worldwide financial markets, particularly after its rapid growth and expansion. The market capitalization of cryptocurrencies reached to 783 billion U.S. dollars till November 2021 (see Figure 1) where it was only closed to 1 billion U.S. dollars in 2013. Parallel to this situation, the significance of cryptocurrency markets on empirical finance has also increased significantly in recent years, gaining considerable interest from academicians, the press, government bodies, financial sector, etc.

Cryptocurrencies are becoming more and more popular in the global financial markets, even though they are highly volatile (Kim et al., 2021). According to Katsiampa (2017), there is a lot of volatility in the price movement of crypto-currencies due to high returns. Bitcoin may be used to hedge against a variety of different risks, including those associated with the stock market, foreign currencies, and commodities (Dyhrberg, 2016). Cryptocurrency can also have a significant impact on stock market (see Vardar and Aydogan, 2019; Salisu et al., 2019). The function of cryptocurrencies relies on the sorts of stock market and time horizons and investment perspectives before it can even be considered secure (Bouri et al., 2017; Shahzad et al., 2020). According to Gil-Alana et al. (2020), investors should diversify their portfolios by including cryptocurrencies into their investments.

Many scholars in recent times explored the correlation between cryptocurrencies and stock market. Several studies (see Conrad et al., 2018; Jiang et al., 2021; Tiwari et al., 2019; Corbet et al., 2018; Salisu et al., 2019) considered advanced economies to explore the impact of cryptocurrency and stock market, provided important insightful stories. Few others (see Lahiani and Jlassi, 2021; Dasman, 2021; Vardar and Aydogan, 2019; Sami and Abdallah, 2020) explored the association in emerging economies. Additionally, the association between cryptocurrency and stock market also received considerable attention among the scholars in the time of COVID-19 (see Mariana et al., 2021; Grobys, 2021; Nguyen, 2021; Kumah et al., 2021). Very few scholars (see Lim and Masih, 2017; Yarovaya et al., 2021) investigated the nexus by considering Islamic stock market. Although several studies empirically investigated the association between cryptocurrency and stock market, a systematic literature review is still important. It is observed that a few numbers of studies, such as Corbet et al. (2019), Rejeb et al. (2021), and Herskind et al. (2020), have performed conventional literature reviews about cryptocurrency since the introduction of the cryptocurrency market. Corbet et al. (2019) investigated the method adopted in earlier research as well as the contributions of earlier scholars and research directions. They also divided the studies into sub-categories based on four study goals: efficiency, bubble dynamics, regulations and cyber-criminality, and bubble dynamics. In the domains of education, accounting, and computers, they comprised quantitative and qualitative investigations published in journals or as working papers. To investigate the obstacles and prospects of cryptocurrencies in modern finance, Rejeb et al. (2021) used a narrative literature review technique. The absence of adequate standards, the potential for illegal activity, governmental prohibitions and use limits, and the extreme fluctuation of cryptocurrencies in modern finance are some of the obstacles they found. A literature study on cryptocurrency and privacy was undertaken by Herskind et al. (2020). They talked about how digital money has changed from electronic currency to cryptocurrencies, with a focus on the ways that users' privacy is protected. Some research (Dabbagh et al., 2019; Jalal et al., 2021) has performed bibliometric analysis. Dabbagh et al. (2019) examined research in the fields of IEEE, computation, and information technology. Jalal et al. (2021) published a bibliometric study on cryptocurrency as a financial asset by considering ISI Web of Science database. Although they considered articles from business, finance, and management domains, the focus was only on the role of cryptocurrencies as a financial asset. Most importantly, the connection between cryptocurrency and the stock market did not receive considerable attention, but rather provided wonderful findings about pricing bubbles, efficiency, diversification and herding behavior, and governance of cryptocurrencies.

Our research differs from past research in a number of ways. First, we only considered the connection between cryptocurrency and the stock market. We also performed both bibliometric and content analysis. Second, our data extends to November 2021, indicating that research undertaken throughout the COVID-19 period (about two years after the initial appearance of COVID-19) is also included. The COVID-19 pandemic changed lots of things in the last few years, and the connection between the stock market and cryptocurrency received significant attention from scholars all over the world. Third, we collected the data from the Scopus database, which has not been considered often in earlier related studies. Scopus is the largest collection of abstracts and citations for peer-reviewed literature covering a broad spectrum of subjects. Thus, the use of Scopus is an effort to cover more subjects that may not be included in WoS (Khudzari et al., 2018).

Therefore, the purpose of this research is to ascertain the state of scientific production about the correlation between cryptocurrency and the stock market. This study contributes to the existing studies by undertaking a more comprehensive review with the purpose of answering the following research questions:

I) What are the most significant features of the literature on the ‘association between cryptocurrency and the stock market,' such as the most productive institutions, countries, authors, journals, authors, and prominent and trending articles and topics?
II) What are the main research strands in the literature on the ‘association between cryptocurrency and the stock market'?
III) What are the future research questions that can be explored regarding the ‘association between cryptocurrency and the stock market’?

This research conducted a systematic review of the literature by collecting data from the Scopus database in the period of 2008 to November 2021, by employing both bibliometric (see Donthu et al., 2021; Xu et al., 2018; Goyal & Kumar, 2021; Zhang et al., 2019; Bahoo et al., 2020; Bhatt et al., 2020; Capobianco-Uriarte et al., 2019) and content analysis (see Nazário et al., 2017; Muresan and Attia, 2017; Geraldi and Ghisi, 2020). Bibliometric analyses enable the identification, organization, and analysis of the major components of a research topic. Additionally, it enables the identification of the most prolific agents in an area of study, whether they are authors, institutions, or nations, which may aid in identifying the agents who are the major driving force behind an area of research (see Bahoo et al., 2020; Bhatt et al., 2020; Capobianco-Uriarte et al., 2019). Thus, this study attempts to make contributions towards existing literature by employing the bibliometric method to explore the most significant features and main research strands in the literature on the relationship between cryptocurrency and the stock market. Most significant features include key journals, most productive countries and organizations, influential studies, and authors. Also, co-occurrence of keywords, co-authorship, and bibliographic coupling are performed.

On the other hand, content analysis is conducted to provide a comprehensive picture about the results and gaps of the prior studies so far. In this section, findings are assessed and discussed based on the major streams. For conducting bibliometric and content analysis, we extracted data from Scopus database. This study conducts a systematic review of 151 papers in total, highlighting the most important features of the literature, the key study streams, and proposing future research questions.

The remainder of the article continues with Section 2, which discusses the evolution of cryptocurrencies over the years. Section 3 presents the structured methodology, including the sample selection, data collection, and analysis procedures for the study. Findings from the bibliometric analysis are illustrated in Section 4. Section 5 contains the discussion of content analysis. Scopes for future research are presented and discussed in Section 6. Section 7 depicts the conclusion of the study.

## 2 Evolution of cryptocurrencies

Just like the internet was a milestone in the growth of communication, cryptocurrency could be the subsequent move in the evolution of finance in this growing electronic era. According to Allen and Bryant (2019), cryptocurrency is the obvious next step in the evolution of currency in a culture that is becoming more electronic, digital, and virtual every day. Although cryptocurrency and its innate technology will become more accessible and useful in the future, it is currently a highly divisive issue (Allen and Bryant, 2019). Although this part is not directly related to our work, it may provide readers with useful information about the history of cryptocurrency. Knowing the evolution of cryptocurrency can be important for this as it is directly involved with the objective (connection between cryptocurrency and stock market). Thus, this section of our study will present a brief overview of the concept and evolution of cryptocurrency based on the literature provided by various scholars.

Different scholars have defined cryptocurrency in their own way. Although there is controversy over the use of 'virtual currency' as a synonym for 'cryptocurrency,' still many of them use some terms including it as alternate for the word ‘cryptocurrency’, such as: 'virtual money,' 'crypto-assets,' 'virtual assets,' 'virtual tokens,' etc. (Стойка, 2021).

According to the European Banking Authority (2014), ‘Virtual Currency’ (VC) is just a digital version of the utility, the only difference being that it is not issued by any government or central bank authority. This digital currency is not tied to a national currency but is meant to be accepted by some individuals as a payment method and can be transferred, saved, and transacted electronically.

Harvey and Tymoigne (2015) defined cryptocurrency as a digital token created using cryptographic algorithms and transferred across cyberspace using protocols. The tokens must have three key features: electronic, not responsible for anybody as well as a peer-to-peer interchange feature (Harvey and Tymoigne, 2015; Bech and Garratt, 2017).

Cryptography originally came from the military and various intelligence agencies that use different codes to protect information from leaking (Bunjaku et al., 2017). However, the technical basis of 'cryptocurrency' or 'virtual currency' dates back to the early 1980s, when American cryptographer David Chaum developed the 'blinding' method (Chaum, 1983). The method is still used in modern web-based encryption (Bunjaku et al., 2017). This secured method of transmitting unchanged and encrypted information through electronic media is the basis of today's electronic fund transfer (Bunjaku et al., 2017). After that, in the late 1990s and early 2000s, the number of digital currencies kept rising, among which some were successful, some not (Bunjaku et al., 2017). For example, Wei Dye's b-money was unsuccessful, but Elon Musk's PayPal has been successful and has become hugely widespread over the years (Arslanian and Fischer, 2019).

However, due to the differences in centralization, and legitimacy with digital currency, in the late 2000s, there was no real cryptocurrency before Bitcoin came into the scene (Bakar et al., 2017). Satoshi Nakamoto (alias) offered Bitcoin for the first time in a white paper published in 2008 (Nakamoto, 2008). In early 2009, he (or they) made Bitcoin accessible to the public (Bunjaku et al., 2017). After that, dozens of cryptocurrencies, including the popular one Litecoin, hit the market by 2010.

**Figure 2:**
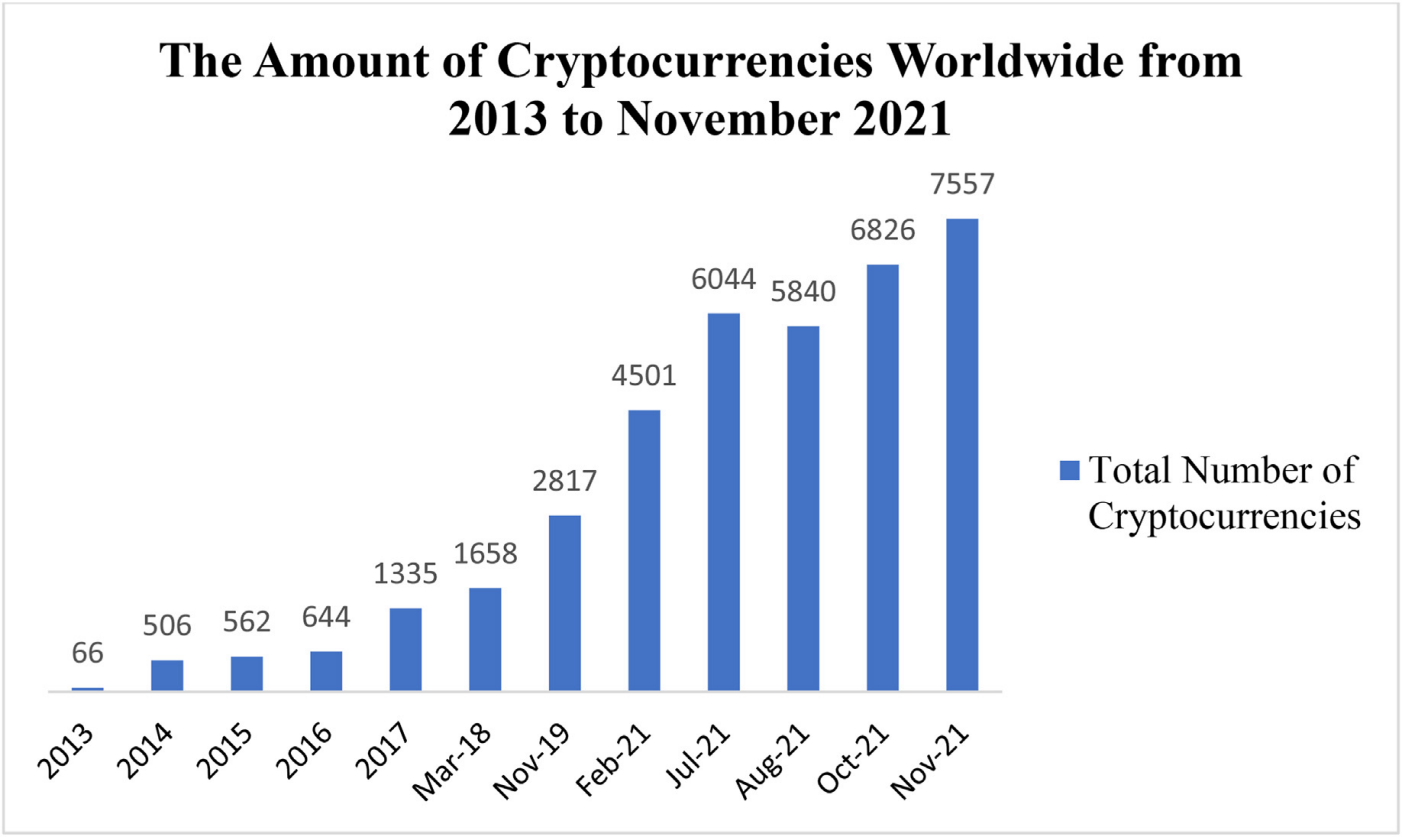
Number of crypto currencies worldwide from 2013 to November 2021.

Statistics (Figure 2) shows a dramatic increase in the number of cryptocurrencies in the last nine years. Just in the last three years, from 2019 to 2021, the total number of cryptocurrencies has increased 2.6 times which is 114 times enhancement compared to 2013.

Despite the significant increase in other crypto-assets, Bitcoin is still one of the most popular cryptocurrencies, almost twelve years after its initial transaction (Söderberg, 2018). In December 2021, from the crowd of cryptocurrencies, the top ten popular cryptocurrencies are Bitcoin (BTC), Ethereum (ETH), Binance Coin (BNB), Tether (USDT), Solana (SOL), Cardano (ADA), XRP (XRP), USD Coin (USDC), Polkadot (DOT), and Dogecoin (DOGE) in terms of market capitalization^1^.

Using a crypto wallet is much like using an ATM card (Jokić et al., 2019). Like bank transactions, cryptocurrency transactions also store user and account information but in an electronic format (Jokić et al., 2019). Every wallet owner has a unique digital address to receive and send cryptocurrencies, still the difference with a bank transaction is that the wallet transaction is sent to an open-source, public ledger (Jokić et al., 2019). The distinction with a back account is here the coin owners get the benefit of encrypted anonymous identity and legitimacy of transaction (Jokić et al., 2019).

According to Vora (2015), cryptocurrencies together with numerous variations are a longed-for evolution to economy because these will compete with prevailing financial and regulatory approaches, stipulate a substitute way for economic agents to transact, and encourage them regards innovation. Impossibility of inflation (because of a preset limit of 21 million Bitcoins), the peer-to-peer crypto-network, unlimited potential for transactions, no boundaries, low operation cost, decentralization, ease of use, confidentiality, and outstanding transaction speed are some common advantages of cryptocurrency (Bunjaku et al., 2017). Unlike traditional bank payment systems, where clients only have access to information about their own accounts, the crypto-currency payment system allows everyone to monitor all other participants' financial transactions, making the system incredibly transparent (Bunjaku et al., 2017). Although sovereign entities do not support cryptocurrencies, Bunjaku et al. (2017) see this transparency of crypto assets as a highly acceptable feature to customers.

However, governing, and central bank authorities in many countries have been vocal in their opposition to the legalization of cryptocurrencies in the financial markets (Harwick, 2016). European Banking Authority (2014) highlighted more than 70 risks of using virtual currencies in their report regarding usage of virtual currencies. Substantial volatility, significant medium-term and long-term risks of investing, risk of financial integration, money laundering, illegal financing, terrorism, and other illicit activities are significant disadvantages of cryptocurrency, which are preventing cryptocurrency from becoming legitimate (European Banking Authority, 2014; Bunjaku et al., 2017).

## 3 Methodology

**Figure 3:**
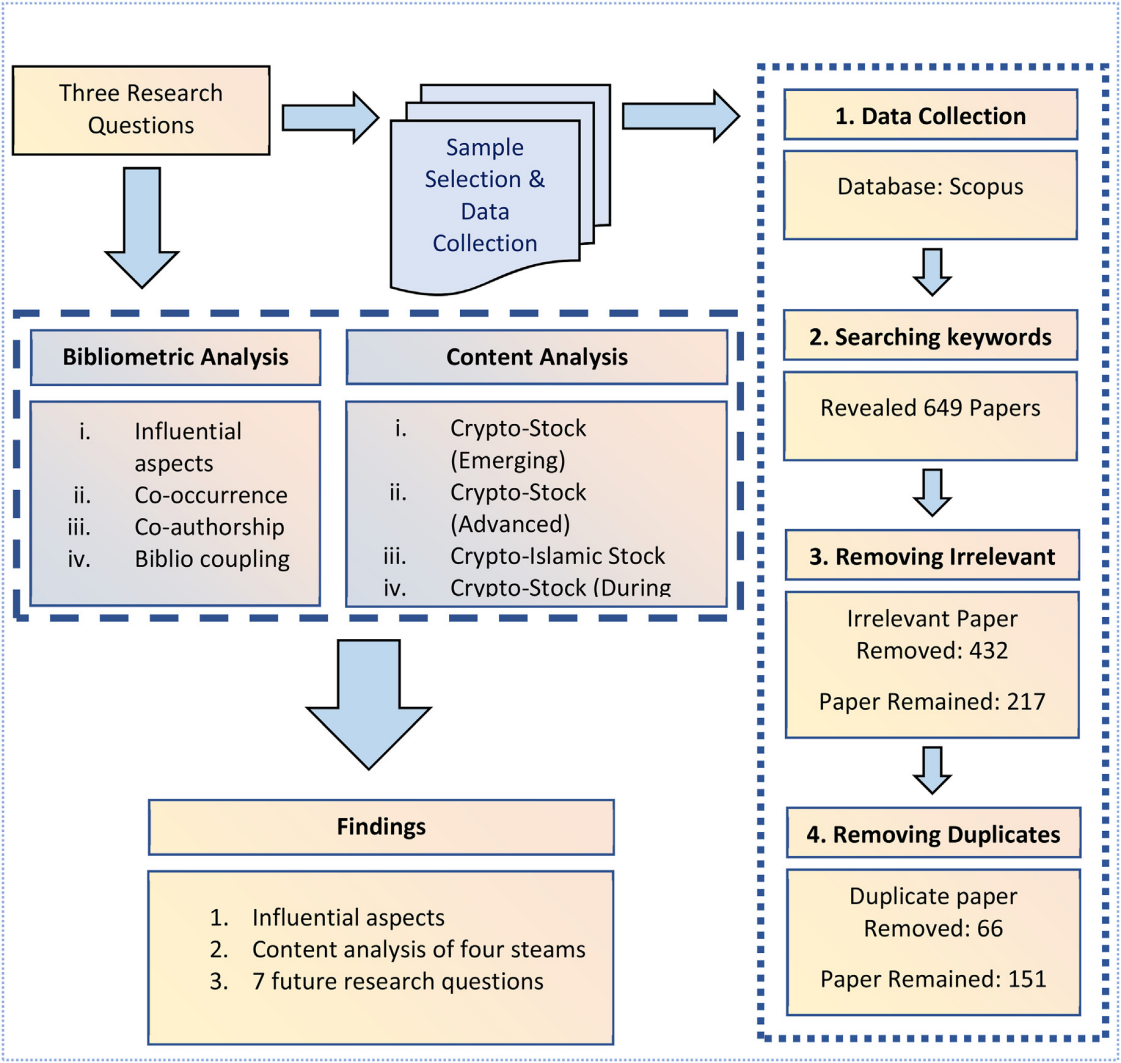
Methodology.

This research used a two-tier analytic approach, integrating bibliometric analysis (see Goyal & Kumar, 2021; Zhang et al., 2019; Bahoo et al., 2020; Bhatt et al., 2020; Chun-Hao and Jian-Min, 2012) and content analysis (see Gelsomino et al., 2016; Kuanova et al., 2021; Nazário et al., 2017; Sharma and Seth, 2012). Bibliometric analysis is a widely used investigation technique that allows researchers to assess the progress of scientific work over the years (Olk and Griffith, 2004; Schildt et al., 2006). Content analysis is a qualitative technique that scholars use to uncover insights about a study's findings and aims (Williamson et al., 2013). In general, content analysis is a widely established approach in the social sciences (Bahoo et al., 2018; Gaur & Kumar, 2018), analyses textual information by shrinking it into more comprehensible groups of data (Weber, 1990). Figure 3 depicts the methodology of the study in detail, including the procedure for sample selection and data collection, as well as the analysis and findings.

### 3.1 Procedure for sample selection and data collection

The data collecting process of this research consists of four parts (see Figure 3). The first approach is to choose a source from which to gather bibliometric data about scholarly papers. This review is based on a gathering of bibliographic data extracted from the Scopus database. Web of Science (WoS) by Clarivate Analytics and Scopus are the two key bibliographic databases (Aria and Cuccurullo, 2017). Scopus indexes nearly 20,000 peer-reviewed journals from many renowned publishers. Web of Science is restricted to 12,000 journals and covers only ISI-indexed journals (Yong-Hak, 2013).

The second step is started with the time period for the sample and the search strategy. A range of keywords are used to look for all articles published between 2008 and November 2021 on the link between cryptocurrency and the stock market. We investigated the following search keywords in combination: (i) "cryptocurrency", "cryptocurrencies", "bitcoin", "AND" (ii) "stock", "stock market", "equity market". It ensures the presence of targeted keywords in extracted research articles. After narrowing the search to include articles from the economics, finance, business, management, computer science, and mathematics sections of Scopus and selecting articles written only in English, 649 articles remained for further consideration.

The third phase involved a detailed assessment of the papers. We reviewed the titles, keywords, and abstracts of the selected articles to remove inappropriate articles. Articles that did not examine the connection between cryptocurrency and the stock market were marked as inappropriate articles. More specifically, the reasons for being left out: they were related to herding in the cryptocurrency market, focused on the evolution of the cryptocurrency market, volatility in the cryptocurrency market, etc. After removing irrelevant articles, we ended up with a dataset of 217 articles. After double checking by each author, duplicate articles were eliminated from the file. In the end, we found 151 research articles that were perfect for figuring out how cryptocurrency and the stock market are related.

### 3.2 Analysis

The analysis of the study consists of two stages: bibliometric and content. In the bibliometric stage, publications and citations trend over the years, influential aspects of the literature, co-occurrence of keywords, co-authorship, and bibliographic coupling are undertaken. We employed diverse tools for statistical and visual processing. We conducted graphs and tables using Excel, network analysis with VOSviewer (Van Eck and Waltman, 2010) and lastly, spatial analysis with mapchart.net. VOSviewer is a more sophisticated tool than CiteSpace and Sci2 for visualizing things using distance-based mapping approaches (Van Eck and Waltman, 2014). While the most of software takes Scopus RIS or WOS files separately, VOSviewer and MS Excel also enabled us to deal with CSV files and integrate data from the two databases. We did a content analysis in the second stage of the analysis and identified four separate research streams based on subject keywords and a thorough analysis of abstracts. Finally, we presented research questions for potential future studies based on the result of the study.

## 4 Bibliometric analysis

### 4.1 Preliminary information on the data

**Figure 4:**
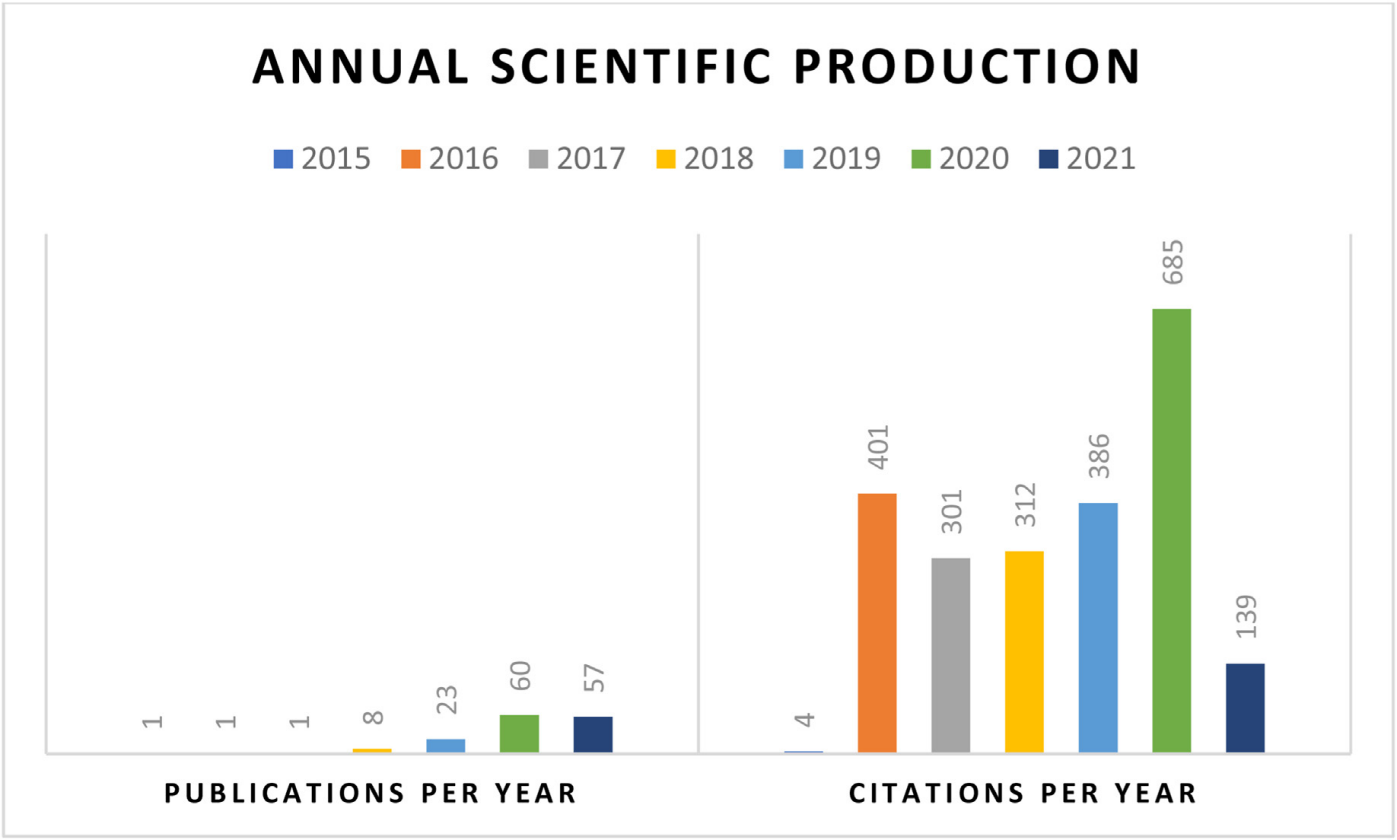
Annual scientific production (Publications and citations per year).

The final sample of 151 publications had 310 authors and were produced in 80 journals, with an average of 14.75 citations per article. Figure 4 presents the total publications and citations related to the association between cryptocurrency and stock market over the years. The number of papers published has been growing, with the most prolific year being 2020, but the number was also high in 2021. Additionally, it is noticed that articles received most citations (around 700) in 2020. The initial trend suggests that in the time of COVID-19, the association between cryptocurrency and stock market received most considerable attention from the scholars.

### 4.2 Influential aspects of the literature

We determined the influential journals, authors, countries, and institutions in the literature related to the association between cryptocurrency and stock market.

#### 4.2.1 Influential journals

The most influential journals were identified using the VOSviewer software. We classified the top five journals into 2 groups: those that published the most articles on the subject (Table 1) and those that received the most citations (Table 2).

**Table 1:** Influential journals (Sorted by publications).

Rank	Journal name	Publications
1	Finance Research Letters	15
2	Research in International Business and Finance	10
3	The North American Journal of Economics and Finance Physica A: Statistical Mechanics and its Applications	6
4	Economic Modelling The Singapore Economic Review The Quarterly Review of Economics and Finance Chaos Solitons & Fractals	4
5	The International Review of Financial Analysis	3

Source: Authors' estimations.

**Table 2:** Influential journals (Sorted by citations).

Rank	Journal name	Citations
1	Finance Research Letters	975
2	Research in International Business and Finance	215
3	Economic Modelling	136
4	Journal of Empirical Finance	106
5	Applied Economics	101

Source: Authors' estimations.

In the case of the highest number of articles published, the most influential journal was Finance Research Letters with 15 articles covering 9.93% of the total publications, followed by Research in International Business and Finance (10, 6.62 The North American Journal of Economics and Finance (6, 3.97%) and Physica A: Statistical Mechanics and its Applications (6, 3.97%). In terms of highest citations received, Finance Research Letters and Research in International Business and Finance again remained top 2 most influential journals with 975 and 215 citations respectively.

#### 4.2.2 Influential authors

The most influential authors of the research are presented in Table 3. Bouri was the most productive author, published 8 articles and received 625 citations. Followed by, Bekiros published 6 documents with 93 citations. Roubaud, Lahmiri, and Jeribi had 5 documents each although Rouband had the highest number of citations (573) among them. Gupta, Shahzad, Kang, Rehman, and Omane-Adjepong all published 3 documents each. Although Gupta published 3 documents, received 3^rd^ highest citations (413 citations).

**Table 3:** Influential authors (Sorted by publications).

Rank	Author	Publications	Citations
1	Bouri, Elie	8	625
2	Bekiros, Stelios	6	93
3	Roubaud, David	5	573
4	Lahmiri, Salim	5	83
5	Jeribi, Ahmed	5	9
6	Gupta, Rangan	3	413
7	Shahzad, Hussain	3	174
8	Kang, Hoon	3	51
9	Rehman, Mobeenur	3	34
10	Omane-Adjepong, Maurice	3	9

Source: Authors' estimations.

#### 4.2.3 Influential countries

**Figure 5:**
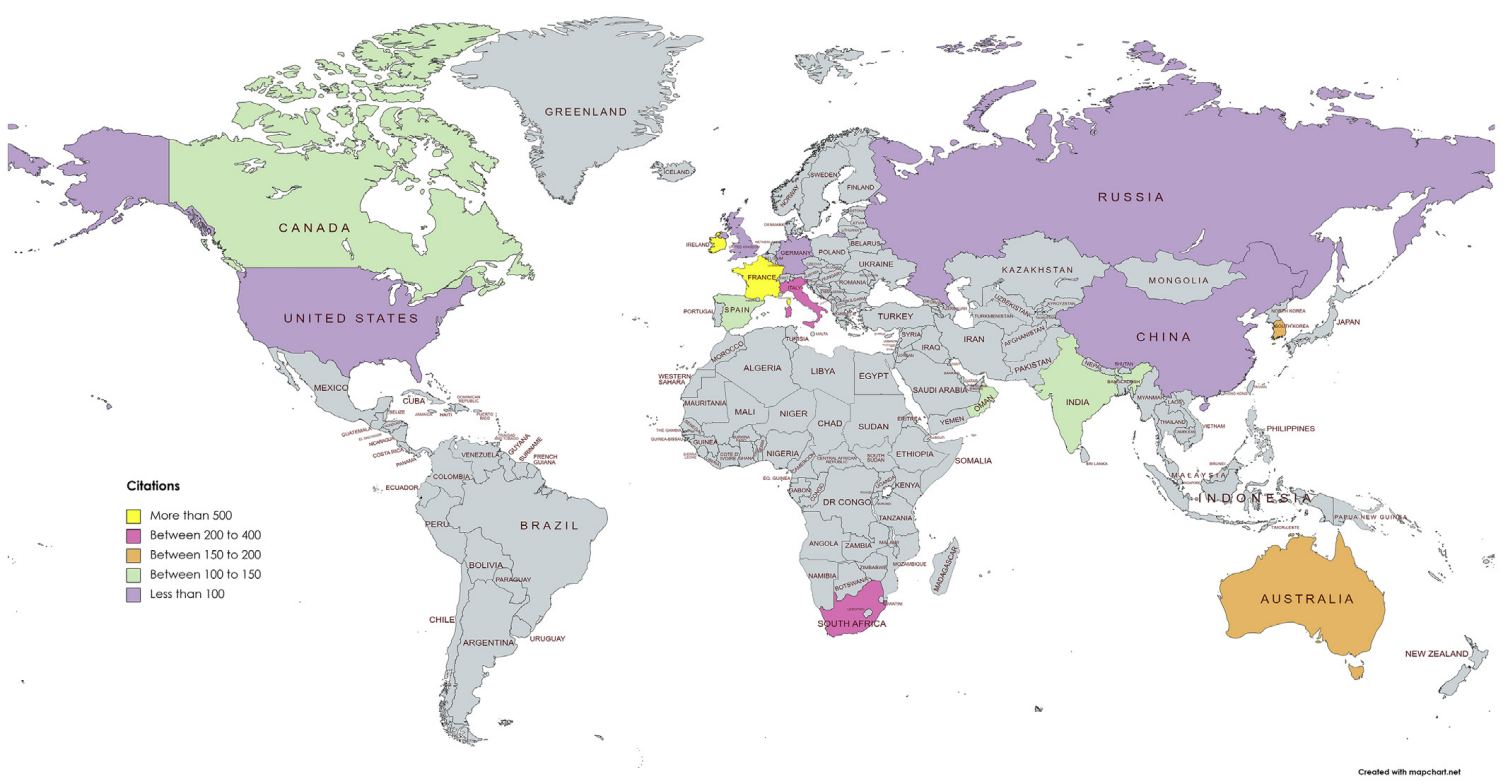
Geographical locations of the study (Sorted by citations).

Table 4 illustrates the top 10 productive countries of the research. China, France, and the United States have made up 26.5% of the total publications. China was the most productive nation with 19 publications, followed by France (11 publications), and United states (10 publications). In terms of highest citations, France was the most prominent (660 citations), followed by Lebanon (625 citations), and South Africa (424 citations). The productive countries are from different regions (Asia, Europe, North America, and Africa) which hints that the association between cryptocurrency and stock market is received good interest around the world. Figure 5 depicts the geographical location of countries based on the total citations received.

**Table 4:** Influential countries (Sorted by publications).

Rank	Country	Publications	Citations
1	China	19	95
2	France	11	660
3	United States	10	72
4	Lebanon	8	625
5	South Africa	8	424
6	Tunisia	8	133
7	Vietnam	8	97
8	Italy	7	200
9	Australia	7	176
10	United Kingdom	7	43

Source: Authors' estimations.

#### 4.2.4 Influential institutions

**Figure 6:**
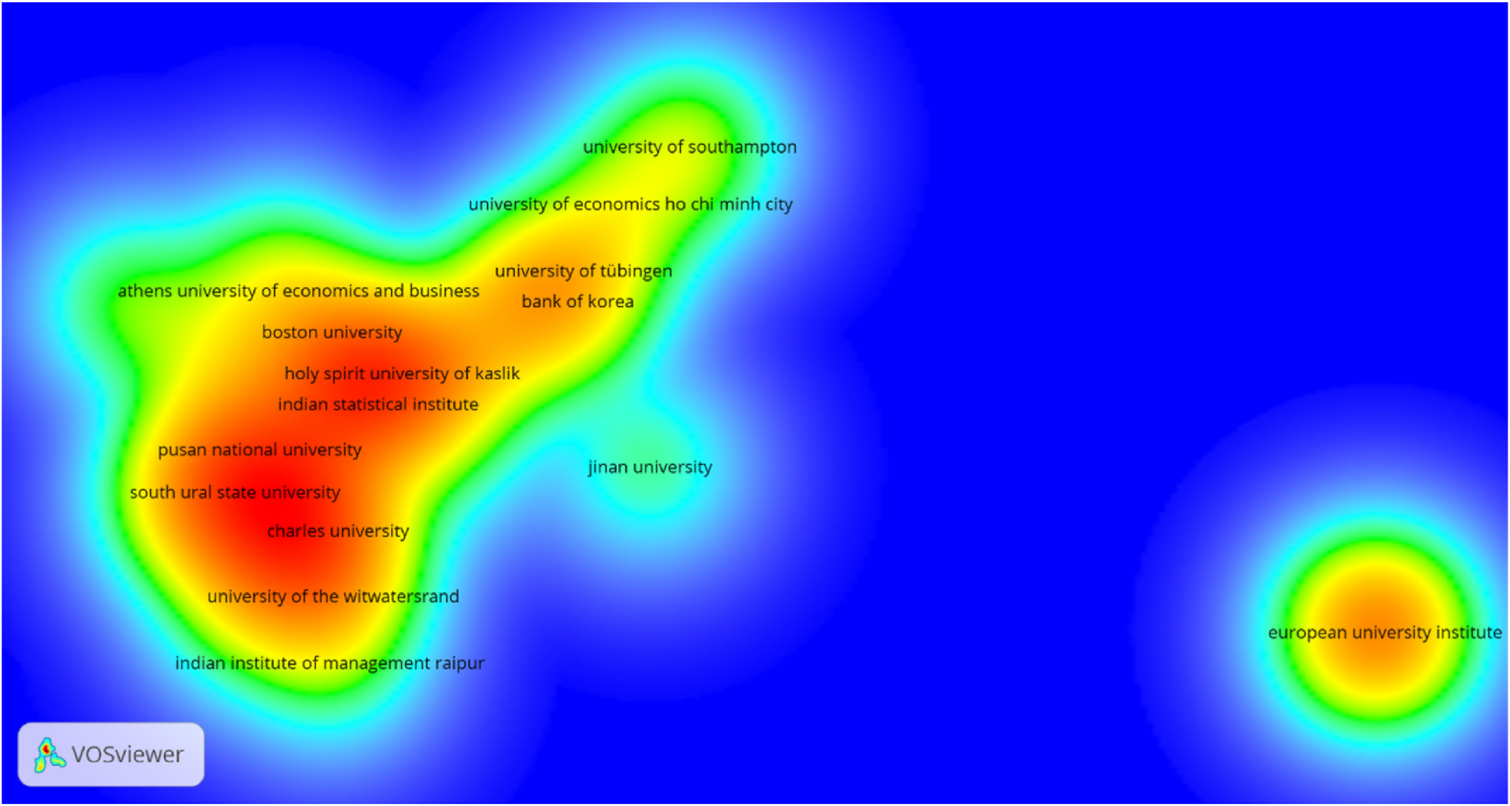
Density visualization of organizations with the highest number of articles.

The most influential institutions involved in the research are presented in Table 5. The four most vital institutions that publish research on the association between cryptocurrency and stock market in terms of the number of contributions Montpellier Business School (five publications), Holy Spirit University of Kaslik (five publications), Pusan National University (five publications), and the European University Institute (five publications). Wilfrid Laurier University, Hunan University, University of the Witwatersrand all have four publications each. In terms of the citations received, Montpellier Business School (599 citations) and Holy Spirit University of Kaslik (597 citations) again shows prominence, followed by the University College Dublin (523 citations), University of Pretoria (413 citations), and Charles University (226 citations). Figure 6 depicts the density visualization of institutions based on the highest number of publications which confirms the influential aspects of the institutions.

**Table 5:** Influential institutions (Sorted by publications).

Rank	Institution	Publications	Citations
1	Montpellier Business School	5	599
2	Holy Spirit University of Kaslik	5	597
3	Pusan National University	5	168
4	European University Institute	5	83
5	Wilfrid Laurier University	4	83
6	Hunan University	4	44
7	University of the Witwatersrand	4	10
8	University College Dublin	3	523
9	University of Pretoria	3	413
10	Charles University	3	226

Source: Authors' estimations.

### 4.3 Co-authorship network among the countries

Co-authorship analysis investigates the interconnections between researchers in a certain area. Since co-authorship is a formal type of intellectual cooperation amongst academics, it is essential to know how scholars engage with one another. In fact, scholarly collaborations may lead to advancements in research; for instance, contributions from several academics might result in better clarity and deeper insights.

**Figure 7:**
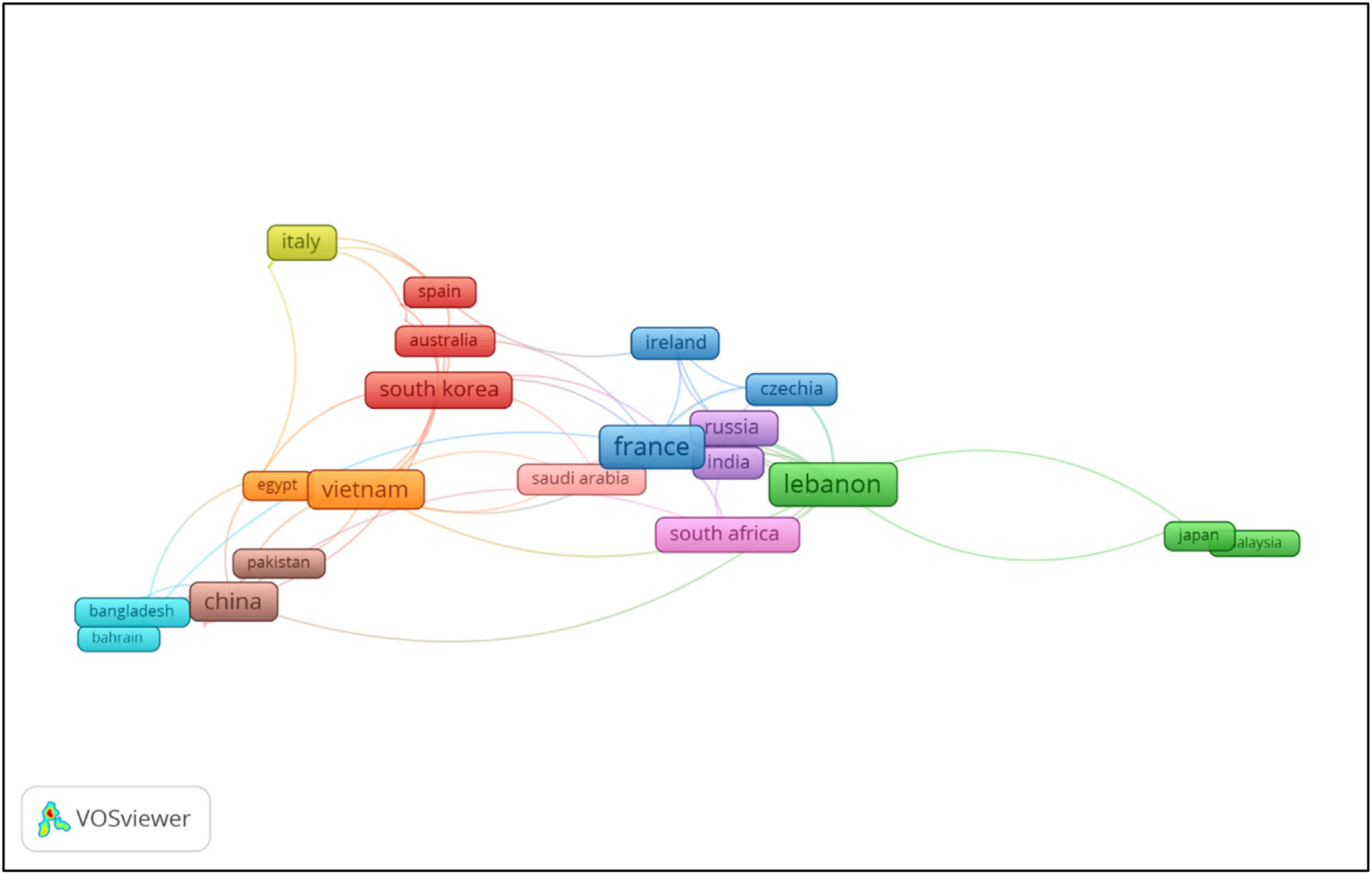
Co-authorship network among the countries.

The study of co-authorship is a significant component of bibliometric study. This section focuses on showcasing the co-authorship network among the scholars and countries through the VOSviewer software. In the context of co-authorship analysis, the link strength between nations denotes the number of publications co-authored by two associated countries, while the overall link strength denotes the strength of a specific country's co-authorship linkages with other countries. Figure 7 depicts the co-authorship network among the countries.

Findings of co-authorship showed that the Lebanon was the most affiliated country, linked to 12 countries with 20 times of co-authorship. The list was followed by France (11 links, 18 co-authorships), Vietnam (11 links, 13 co-authorships), China (10 links, 13 co-authorships), South Korea (9 links, 10 co-authorships), and others. Overall, authors from Lebanon showed the most interest in this research issue.

### 4.4 Bibliographic articles coupling

**Figure 8:**
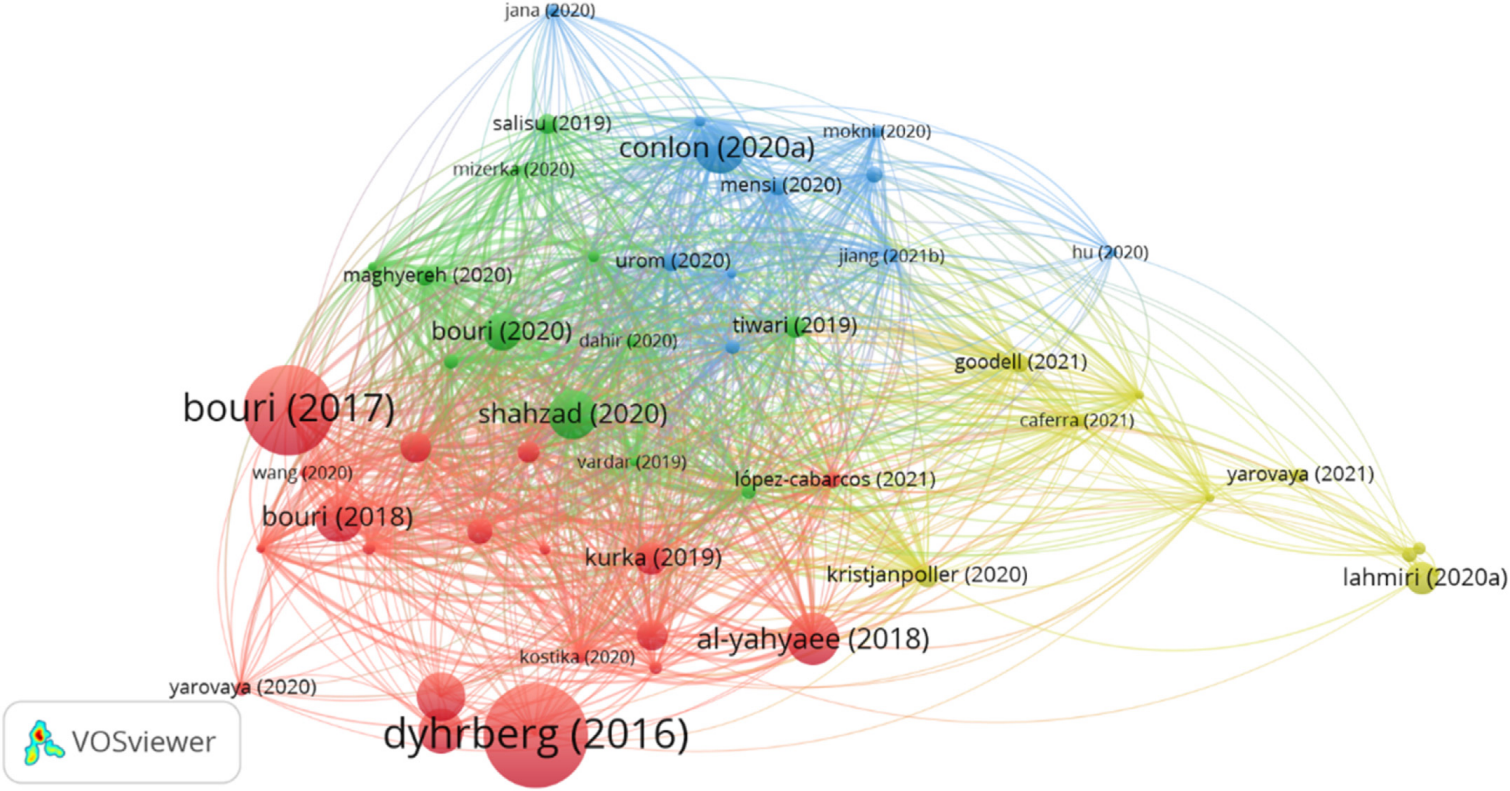
Bibliographic coupling of articles.

The principal goal of bibliographic coupling is to determine the connection between the citations of individual papers and to identify the clusters and themes covered within the database. This test was conducted using VOSviewer. Figure 8 displays the bibliographic coupling of papers pertaining to cryptocurrencies and the stock market, which is divided into four separate clusters. After VOSviewer had identified the clusters, each author manually reviewed the title, abstract, research question(s), theory, data sources, variables, and results of each article. This content analysis allowed us to get an overview of the findings and contributions of each paper, which ultimately made it easier to finalize the streams appropriately. As a result, we identified four research streams in the literature, i) cryptocurrency and stock market in emerging countries, ii) cryptocurrency and stock market in advanced countries, iii) cryptocurrency and Islamic stock market, and iv) cryptocurrency and stock market during the COVID-19.

We found some papers examined the connection between cryptocurrency and stock market by considering only emerging countries. The same case went for advanced countries. After critically reviewing the articles, we also found some articles that provided attention only to Islamic markets. Although, it is niche, we feel it can be presented separately as a few papers considered important to explore. In the time of COVID-19, some articles examined the nexus, and the results were different from those in the pandemic-free time. We believe that presenting it separately will provide readers with an overview of the scenario we are witnessing during COVID-19, allowing us to fight any future pandemic.

Overall, sections 4.2.-.4 provided useful findings. In the 4.2 section, we identified the influential aspects (journals, authors, countries, and institutions) of the literature on the basis of the number of publications and the number of citations. This allowed us to understand the intellectual dynamics of the issue by identifying the most influential aspects of the literature. The fact that journals and academics continue to use social media to promote research is another reason for its importance. Thus, identifying influential aspects is vital. In Section 4.3, a co-authorship network among the countries was identified. This enabled us to understand the status of collaborations between countries (authors’ nationality) on this research issue. Bibliographic article coupling (section 4.4) assisted us in finding the major clusters. After that, we identified the major streams through a critical content analysis.

## 5 Content analysis

### 5.1 Relationship between cryptocurrency and stock market in emerging countries

The relationship between cryptocurrency and stock market has allured considerable attention of everyone participating in the market, from capitalists to bankers, entrepreneurs, and political circles. Scholars have been trying to find a pattern on this association over the years. Researchers have found a multifaceted relationship of cryptocurrency with stocks depending on the country's erratic inflation and fast-moving exchange rate, complex and expensive banking system, regulatory uncertainty, financial constraints, and especially the existence or threat of capital control. This part of the paper summarizes the prior research done by researchers on cryptocurrency and stock relations in emerging countries.

According to the MSCI Marketing Index, emerging markets consist of developing nations that are rapidly growing and industrializing^2^. In 2021, this market includes 25 countries and some of these countries fall into a few economic blocks, such as BRICS, CIVETS, BEM, and MENA^3^. To depict a clear picture, the prior research findings are exhibited here by the segment of blocks. Table 6 presents some key findings regarding the association between cryptocurrency and stock market in the emerging market.

**Table 6:** Key findings regarding the correlation between cryptocurrency and stock market in emerging countries.

Author	Cryptocurrency Used	Country	Main Findings
Wang et al. (2019)	973 cryptocurrencies	30 international indices is used	• Stock markets of Brazil, China, and Japan are the most instable and unpredictable compared to the developed markets
Lahiani and Jlassi (2021)	Bitcoin, Dash, Ethereum, Monero, Ripple	BRICS and Developed Countries	• No substantial correlation exists between cryptocurrencies and the stock market of BRICS • Crypto-stock relations have improved since the introduction of the 'Bitcoin Future' concept in 2017. • In the BRICS economy, only Brazilian stock shows a relationship with the cryptocurrencies (Dash, Ethereum & Monero
Jeribi and Ghorbel (2021)	Bitcoin, Dash, Ethereum, Monero, and Ripple	BRICS and Developed Countries	• Bitcoin does not play a hedging role in BRICS countries compared to developed nations • Only Bitcoin has a dynamic, positive correlation with the Brazilian and South African stock market.
Kumah and Odei-Mensah (2021)	Bitcoin, Ethereum, and Litecoin	Africa (Egypt, South Africa, Nigeria, Mauritius, Kenya, Ghana, Tunisia, and Morocco)	• Bitcoin has a medium-term integration with Egyptian stocks market. • In the long run, Ethereum exhibits a strong negative, but Litecoin demonstrates a positive effect on the Egyptian market.
Vardar and Aydogan (2019)	Bitcoin	Turkey	• Bilateral cross-market shock and volatility spillover impact exists between bitcoin and Turkish stock market
Omane-Adjepong et al. (2021)	8 cryptocurrencies (Bitcoin, Ethereum, Ripple, Litecoin, Dash, Ethereum Classic, NEO, and Zcash)	G20	• Asymmetric herd behavior exists between cryptocurrencies and stock markets of 10 emerging economies

#### 5.1.1 BRICS

BRICS consists of the five major countries (Brazil, Russia, India, China, and South Africa) of the emerging markets. By observing 973 types of cryptocurrencies from 30 global indices, Wang et al. (2019) found the stock markets of Brazil, China, and Japan are the most instable and unpredictable compared to the developed markets. Furthermore, they used the DCC-GARCH model and found no cryptocurrencies as a ‘Safe Haven’ for BRICS market (Wang et al., 2019).

Lahiani and Jlassi (2021) and Jeribi and Ghorbel (2021) independently analyzed the correlation of the top five leading cryptocurrencies (Bitcoin, Dash, Ethereum, Monero, Ripple) with the BRICS market and advanced economy, but none of them found any substantial correlation between cryptocurrencies and the stock market of BRICS compared to advanced economy. With few exceptions, before 2017, when the concept of "Bitcoin Future" was introduced, no cryptocurrency had the mean power of predicting the stock market (Lahiani and Jlassi, 2021). However, with the advent of the notion of the future of Bitcoin, the picture of the crypto-stock relationship has changed, although, in the BRICS economy, only Brazilian stock shows a relationship with the cryptocurrencies (Dash, Ethereum & Monero), which is quite negligible association against developed countries. In addition of Brazilian stock, Jeribi and Ghorbel (2021) found a dynamic, positive correlation of only Bitcoin with South African stock.

Bitcoin does not play a hedging role in BRICS countries compared to developed nations; instead, it is found as a diversified asset for this emerging market (Jeribi and Ghorbel, 2021). Jeribi and Ghorbel (2021) added that, for this economy, Bitcoin could be thought of as the new gold. To sum up, it is perceived that most researchers have illustrated the effect of several cryptocurrencies on the stocks of both developed and emerging countries together and comparatively BRICS economy had a lower correlation. And the result is a slight impact of cryptocurrencies other than Bitcoin is found on this emerging economy.

#### 5.1.2 CIVETS and Middle East and North Africa (MENA)

After the BRICS market, the most vital emerging market is CIVETS (Colombia, Indonesia, Vietnam, Egypt, Turkey, and South Africa)^1^. The economic importance of these countries is expected to increase rapidly in the coming days^1^.

Bitcoin has a medium-term positive integration with Egyptian stocks market (Kumah and Odei-Mensah, 2021). In the long run, Ethereum exhibits a strong negative, but Litecoin demonstrates a positive effect on the Egyptian stock market (Kumah and Odei-Mensah, 2021). Except for the US dollar exchange rate, there is ample evidence of the impact of the bilateral cross-market shock and volatility spoil over on the Turkish market between Bitcoin and all other financial asset classes (Vardar and Aydogan, 2019).

#### 5.1.3 Others

There are some other regions or emerging countries where researchers emphasized investigating the connection between cryptocurrencies and the stock market. This part will discuss some of their findings.

Hung (2021) found that Bitcoin has an average positive correlation with the stock market of CEE (Croatia, Hungary, Poland, Romania, and the Czech Republic), using the Dynamic Equi-correlation GARCH (DECO-GARCH) model. Omane-Adjepong et al. (2021) found an asymmetric herd behaviour in the cryptocurrency and stock markets of 10 emerging economies within G20 conducting the cross-sectional absolute deviation (CSAD) approach. Philippines and Thailand's stock market has a positive but Malaysian stock market has an adverse relationship with cryptocurrency (Thampanya et al., 2020).

### 5.2 Relationship between cryptocurrency and stock market in advanced countries

The dynamic association between cryptocurrency and stock markets has been the focus of numerous research work in advanced economies. Studies have found plausible reasons behind the existence of this relationship. This subsection will discuss the interconnection between cryptocurrencies and stock market indices of advanced economies. Table 7 presents some key findings regarding the association between cryptocurrency and stock market in advanced countries.

**Table 7:** Key findings regarding the correlation between cryptocurrency and stock market in advanced countries.

Author	Cryptocurrency Used	Country	Main Findings
Erdas and Caglar (2018)	Bitcoin	USA	• Causal relationship exists.
Salisu et al. (2019)	Bitcoin	G7 countries	• Stock market returns in the G7 have a strong positive correlation to cryptocurrency's predictability.
Wang et al. (2019)	Bitcoin	Europe, USA	• A significant time-of-day and bilateral causality relation exist between the momentary variables of Bitcoin and global equity markets.
Zhang et al. (2018)	Bitcoin, Ethereum, Ripple	USA	• The association of Bitcoin and traditional assets is minimal, and it ​fluctuates.
Zhang et al. (2021)	Bitcoin	USA	• There is a time-dependent investment volatility spillover that subsists between bitcoin and four other ​commodities.
Isah and Raheem (2019)	Bitcoin	USA	• Forecasting programs based on BTC are far more effective at predicting equity returns.
López-Cabarcos et al. (2021)	Bitcoin	USA	• As per the findings, Bitcoin instability is more unpredictable during volatile times.
Wang et al. (2020)	Bitcoin	USA	• The S&P 500 does have a moderate impact on Bitcoin, whereas the effect of ​the S&P 500 is diluting.
Tiwari et al. (2019)	Litecoin	USA	• Litecoin is indeed the safest way to hedge over the US ​equity market's volatility.
Wang et al. (2021)	Bitcoin, Ethereum	USA	• Asymmetric contagion effects between the two financial markets.
Charfeddine et al. (2020)	Bitcoin, Ethereum	USA	• The connection between cryptocurrencies and traditional assets is highly vulnerable to ﬁnancial and economic ​disruptions of the outside world.
Corbet et al. (2018)	Bitcoin, Ripple, and Litecoin.	USA	• Cryptocurrencies could be a good way for tight speculators to expand their investment opportunities.

Isah and Raheem (2019) explored the theoretical predictive ability of cryptocurrencies that may be implicit in US stock prices. They documented that the BTC-based predictive models are precise in forecasting stock returns. Furthermore, they mentioned that predicting stock returns is more precise and viable when the underlying reasons, such as quantitative easing which have sustained the growth of cryptocurrency are considered. Erdas and Caglar (2018) analyzed the impact of bitcoin on the US stock index using the asymmetric causality test. Their result indicates that the linkage between bitcoin and the S&P 500 Index is unidirectional. The findings also imply that a negative shock in bitcoin induces negative and positive shocks in the S&P 500 Index, whereas a positive shock in bitcoin causes negative shocks in the S&P 500 Index. There is also some other evidence to suggest that the S&P 500 Index is correlated to bitcoin (Conrad et al., 2018; Chan et al., 2019). The median and tail dependence between cryptocurrency and stock market returns of advanced countries have been investigated by Lahiani and Jlassi (2021) in which they have applied Generalized Auto Regressive Conditional Heteroskedasticity (GARCH) and subsample analysis. The result of the study demonstrates that in both the mean and tails, the correlations between cryptocurrencies and stock markets have been affected by the introduction of bitcoin futures. For instance, prior to the start of Bitcoin futures, cryptocurrencies did not have a strong mean predictive ability in projecting stock market returns. Conversely, in the timeframe following Bitcoin futures, cryptocurrencies have the extra-strong predictive ability in projecting equity market returns, explaining the depth of tail dependence between the two financial markets. Additionally, the study revealed that Ethereum, followed by bitcoin, plays the pivotal function in forecasting cryptocurrencies and stock market returns in advanced economies. In another different study, the effect of bitcoin prices in forecasting stock returns for G7 countries has been examined (Salisu et al., 2019). By applying the baseline predictive model, the researchers said that there is a considerable positive connection exists between the predictability of bitcoin and stock returns of developed countries. However, except for Japan, the BTC-based model estimates the stock returns of the G7 economies substantially better than their macroeconomic factors altogether. Based on five developed countries, Jiang et al. (2021) carried out a study on Bitcoin, Ethereum, Stellar, and Monero using the Quantile Coherence Matrix model, and they revealed that cryptocurrencies behave as weak hedges or secured places for equity markets. Litecoin, on the other hand, has a negative association with Japan's stock market index. Illustrating that, in the short run, Litecoin might perform as a stable, secured reserve for the stock market index of Japan when the overall economy is witnessing a downturn. The consistently evolving dependent relationships between cryptocurrencies and stock market indexes of Japan and the USA are largely positive, signifying that stock markets are inclined to acquire price fluctuations in cryptocurrency markets (Umar et al., 2020). In a recent study, Wang et al. (2021) evaluated the asymmetric contagion impact within the US stock market and the cryptocurrency market and hypothesized that interactive linkages exist in both these two forms of financial marketplaces. Moreover, they explained that the cryptocurrency market's price changes could serve as a "weathervane" for predicting stock market price fluctuations. A time-varying asymmetric link between cryptocurrencies and the US stock market was examined by Tiwari et al. (2019), and the findings disclosed that Litecoin is the best efficient hedging financial attribute in opposed to the volatility of the US stock market. A one-way causality was observed between bitcoin and Nikkei225, S&P500 of Japan and the United States, respectively (Ünvan, 2021). By exploring the association between bitcoin and the equity market and applying a vector autoregressive model (VaR), Wang et al. (2020) pointed out that the S&P 500 has a larger impact on bitcoin whereas the influence carried out by the S&P 500 is relatively insignificant. Additionally, they argued that the S&P 500 and the Dow Jones indices have a positive influence on bitcoin. Zhang et al. (2021) observed time-dependent downside risk spillover between bitcoin and the US stock markets while investigating the relationship between these two financial markets.

Tiwari et al. (2019) document the weak favorable connection between each cryptocurrency and S&P 500 index. In another different study, López-Cabarcos et al. (2021) analyzed the effect of the US stock market on bitcoin volatility applying Generalized Auto Regressive Conditional Heteroskedasticity (GARCH) and Exponential Generalized Auto Regressive Conditional Heteroskedasticity (EGARCH) models, and they narrated that bitcoin fluctuation is turbulent in speculative cycles. In the same vein, Bouri et al. (2017) assert that the tentative aspect of cryptocurrencies may enhance risk contagion between stock and cryptocurrency markets, lowering the former's hedging and relatively secure haven capacity in the brief term. By using the approach of generalized variance decomposition implied by Diebold and Yilmaz (2012) and, Corbet et al. (2018), they disclose that cryptocurrencies are not affiliated with stock indices. Ever since several researchers have indicated similar conclusions using various GARCH models. (e.g., Charfeddine et al., 2020).

### 5.3 Relationship between cryptocurrency and Islamic stock market

Over the years, several studies have been undertaken regarding the relationship between cryptocurrency and traditional stock market. Unfortunately, Islamic stock market was not as focused as the regular stock market. As a result, we realized the need of discussing the connectedness between cryptocurrencies and Islamic stock markets in our research, which will benefit future investors and expand the literature resources on this subject. Table 8 presents some key findings regarding the association between cryptocurrency and Islamic stock market.

**Table 8:** Key findings regarding the correlation between cryptocurrency and Islamic stock market.

Author	Cryptocurrency Used	Country/Stocks	Main Findings
Rehman et al. (2020)	Bitcoin	DJIJP, DJICA, and DJIUK	• Bitcoin has time-varying dependence only with DJIJP, DJICA, and DJIUK among all the major Islamic indices.
Mensi et al. (2020)	Bitcoin	DJIM, IMUS, DJIEU, DJIAP, DJIUK, DJIJP, DJICA, IMXL, DJSUKUK	• Long-term investments in Islamic equity markets may generate less diversification benefits than short-term investments.
Ahmed (2021)	Bitcoin	Developed economics	• Bitcoin's upward volatility has contemporary and lagged negative impacts on Islamic indices more in bear (downward) than bull (upward) trending markets. Meanwhile the downward volatility seems to have a significant effect on the returns if Sharia-compliant stocks are on a downward and upward trend.
Yarovaya et al. (2021)	Bitcoin	DJIM, DJIA, ICE BofA Global, DJSUKUK	• COVID-19, gold and oil are important predictors of traditional-Islamic markets spillovers, but Bitcoin is not a crucial predictor.

Bitcoin has time-varying dependence only with DJIJP, DJICA, and DJIUK among all the major Islamic indices. Additionally, the results also revealed that there was an existence ​of risk spillover among Bitcoin and Islamic stock markets. Moreover, Islamic stock market performs as a useful hedge in a portfolio together with Bitcoin (Rehman et al., 2020).

Mensi et al. (2020) discovered relatively close but more specific information than (Rehman et al., 2020) that long-term investments in Islamic equity markets may generate less diversification benefits than short-term investments. Thus, the advantages of diversifying portfolios alongside Bitcoin and Islamic commodities differ based on the time and frequency. According to Ahmed (2021), in developed economies, Bitcoin's upward volatility has contemporary and lagged negative impacts on Islamic indices more in bear (downward) than bull (upward) trending markets. Meanwhile the downward volatility seems to have a significant effect on the returns if Sharia-compliant stocks are on a downward and upward trend.

Furthermore, a recent investigation by Yarovaya et al. (2021) unveiled that COVID-19, gold and oil are important predictors of traditional-Islamic markets spillovers, but Bitcoin is not a crucial predictor. The study also suggested that traditional-Islamic assets spillovers are determined by very few factors where oil and gold prices remaining the most significant. However, Bitcoin failed to establish the connection between traditional and Islamic markets.

### 5.4 Relationship between cryptocurrency and stock market during the COVID-19

The connection between cryptocurrencies and stock market in advanced and emerging countries has received tremendous consideration over the years. Many scholars explored the association by considering both conventional stock market and Islamic stock indices. In the time of pandemic, the whole scenario may change dramatically as lots of factors become vulnerable and for this reason, several researchers investigated the nexus between cryptocurrencies and stock market during the time of COVID-19 turmoil. This section will discuss findings related to the association between cryptocurrencies and stock market during the time of COVID-19. Table 9 presents some key findings regarding the association between cryptocurrency and stock market during the time of COVID-19.

**Table 9:** Key findings regarding the correlation between cryptocurrency and stock market during the COVID-19.

Author/s	Cryptocurrency Used	Sample (Countries)	Key Findings
Jeribi et al. (2021)	Bitcoin, Dash, Ethereum, Monero, and Ripple	BRICS (Brazil, Russia, India, China, and South Africa) stock markets	• During the COVID-19 financial crisis, Bitcoin, Ethereum, Dash, Monero, and Ripple were discovered to be a safe haven for three developing economies (Brazil, China, and Russia).
Lahmiri and Bekiros (2021)	45 Cryptocurrency markets	16 International Equity markets	• The pandemic of COVID-19 has had a substantial impact on the long-term return and volatility of cryptocurrencies and international stock markets.
Umar and Gubareva (2020)	Bloomberg Galaxy Crypto Index (BGCI)	EUR, GBP, RMB	• All PI-currency pairings exhibit comparable patterns over time and frequency scales in their individual heatmaps, revealing a high degree of coherence and dependency around the COVID-19 panic's apogee in mid-March.
Conlon et al. (2020)	Bitcoin, Ethereum and Tether	US (S&P 500), UK (FTSE 100), Italy (FTSE MIB), Spain (IBEX), and China (CSI 300)	• Bitcoin and Ethereum are not a safe haven currency for the vast majority of foreign equities markets assessed during the course of the covid-19 era. • Tether is found to act as a safe haven over the most recent period including the COVID-19 crisis.
Mariana et al. (2021)	Bitcoin and Ethereum	USA (S&P500)	• Bitcoin and Ethereum are appropriate as safe havens for the near term. • Ethereum is preferable than Bitcoin as a safe haven when the stock market goes down quickly. • Ethereum has a higher rate of return volatility than Bitcoin.
Corbet et al. (2020)	Bitcoin	China (CSI 300)	• Gold and other cryptocurrencies don't have a big impact on the Chinese stock market as assessed by the price of Bitcoin in China.
Grobys (2021)	Bitcoin	USA (S&P500)	• The drop in the price of Bitcoin was not just caused by the COVID-19 pandemic. It was also caused by problems with cryptocurrency exchanges' market microstructure.
Yousaf and Ali (2021)	Litecoin, Bitcoin, and Ethereum	USA	• During the pre-COVID-19 era, return overflow and instability between the US stock market and the crypto market were low.
Caferra & Vidal-Tom´as (2021)	Bitcoin and Ethereum	USA and Eurozone (France and Germany)	• Both cryptocurrency and stock values plummeted precipitously during COVID-19. Despite this correction, cryptocurrencies immediately recovered, while the stock market remained in bad territory.

Jeribi et al. (2021) used the Nonlinear Autoregressive Distributed Lag (NARDL) technique and claimed that the dynamic relationship among cryptocurrency return and stock market return remaining between both short and long-run has changed during the COVID-19 pandemic. Moreover, the authors also noticed that Bitcoin, Ethereum, Dash, Monero, and Ripple are haven for Brazil, China, and Russia (emerging stock markets) during the COVID-19 economic crisis. However, Mariana et al. (2021) analyzed the connection between S&P500 and two cryptocurrencies (Bitcoin & Ethereum) by using the DCC-GARCH technique claimed that Ethereum shows higher profit volatility than Bitcoin and is a better shed than Bitcoin during the COVID-19 pandemic when the stock market was facing downturn. Moreover, the authors also opined that both cryptocurrencies are appropriate as short-term safe heavens. On the contrary Conlon et al. (2020) noticed that neither Bitcoin nor Ethereum is a secure haven, but Tether acted as a secure haven for the global stock markets (FTSE 100; S&P 500; IBEX; FTSE MIB and CSI 300) during COVID-19 turmoil.

Kumah et al. (2021) found weak interdependence between markets during the COVID-19 crisis. He also stated that for African stocks and commodity exponent both Ethereum and Tether are safe havens in medium term. Considering 16 international equity markets and 45 cryptocurrency markets, Lahmiri and Bekiros (2021) opined that the COVID-19 turmoil has considerably influenced long term memory reciprocally and cryptocurrency's volatility along with the global stock markets. Umar and Gubareva (2020) observed the COVID-19 pandemic effect on the volatility of the currency and crypto markets using the time-frequency analysis. They found that all crypto pairs showed similarity along time and frequency scales in their respective heatmaps and implied high consistency and interdependency around the mid-March when COVID-19 was peak.

Grobys (2021) studied the progressive correlation between US stocks and Bitcoin. He then explored that the Bitcoin price drop was not only due to the COVID-19 pandemic but also because of the issues with the microstructure of the crypto exchange market. Alongside this, Bitcoin performed poorly in hedging during the COVID-19 pandemic crisis (Grobys, 2021). Using standard GARCH, Corbet et al. (2020) pointed out that neither gold nor cryptocurrency have significant associations with the Chinese stock market as measured by Bitcoin price movements. However, analyzing this same using high-frequency data, the authors argued for a unique interrelation between Chinese stock markets at the beginning of the COVID-19 pandemic.

Yousaf and Ali (2021) have conjointly performed a very recent investigation and detected that during the pre-COVID-19 period the return spillover and volatility between the crypto market and the US stock market were negligible. The authors also found that during the COVID-19 period S&P 500 to all the cryptos had a one-way reverse transfer. The volatility spillover was unidirectional from S&P 500 to Litecoin, whereas the volatility propagation is insignificant for S&P 500–Ethereum and S&P 500–Bitcoin pairs during the COVID-19 period, Nguyen (2021) reviewed and stated that financial market thrust during the COVID-19 turbulence affected Bitcoin's volatility. In addition, the author also stated that stocks and cryptocurrency markets are more interconnected in times of uncertainty.

Caferra & Vidal-Tom´as (2021) stated their research results showed a financial contagion in March during COVID-19 when both cryptocurrency and stocks plummeted. Despite this downturn, cryptocurrencies rebounded quickly while the stock market was stuck in a bearish phase.

## 6 Future research questions

The literature on the relationship between cryptocurrency and the stock market is expanding rapidly. However, there are still vital issues to investigate. The combination of bibliometric analysis and content analysis enables us to propose several directions for further study. After selecting papers from bibliometric analysis, the authors critically reviewed the contents (abstract, findings and discussions, and conclusions) of the papers. Following that, we converted research questions from future study agendas into research questions. We have also identified future research questions by finding gaps in the literature that are not mentioned in the previous studies. Table 10 presents the possible research questions for future investigations. .

**Table 10:** Future research questions.

Sl. No	References	Research Questions/Explanations
1	Authors' suggestion	Does the correlation between Bitcoin return volatility and socially responsible indices have any possible utility for stock investors in terms of portfolio diversification? What will be the major discoveries if various aspects, including macroeconomic variables and fundamental factors are considered?
2	(Tiwari et al., 2019)	What significance does cryptocurrency have in hedging against other financial assets like bonds, currency, and uncertainty?
3	(Jiang et al., 2021)	How do different cryptocurrencies role the stock markets? Does “Bitcoin Futures” as an added derivative impact the role of cryptocurrencies?
4	(Omane-Adjepong et al., 2021)	Do internal and/or external factors influence the herding behavior between cryptocurrency and emerging markets?
5	(Zhang et al., 2021)	Will assessing the risk spillover between Bitcoin and disaggregated stock indexes provide any significant outcome? What if Ethereum and Ripple are utilized combining with Bitcoin to better reflect the global cryptocurrency markets? Would the results be any different?
6	Authors' suggestion	What are the impacts of cryptocurrencies other than Bitcoin on the stock market?
7	(López-Cabarcos et al., 2021)	Is it possible that Bitcoin and investor sentiment have a bidirectional connection? Does analyzing the correlation between Bitcoin and other financial factors such as, currencies & indices convey any significant ideas for future investors?

Firstly, the authors’ suggested to include as much as Islamic index to find a comprehensive finding. It is also important to analyze the correlation of Bitcoin return volatility with socially responsible indices replacing the Islamic stock indices which could generate significant findings for stock investors in terms of portfolio diversification. Furthermore, there could be some major ideas if various aspects, including macroeconomic variables and fundamental factors are analyzed.

The role and significance of cryptocurrency in hedging against other financial assets like bonds, currency, and uncertainty can be another important area for further research. Tiwari et al. (2019) mentioned that exploring the role in other financial assets can give the relative bodies some important idea to choose the better financial assets. More specifically, the finding from these areas will extremely help the investors to diversify their portfolios.

Jiang et al. (2021) analyzed the role of multiple cryptocurrencies with the renowned stock indexes and recommended the future researchers to extend the study by adding other kinds of cryptocurrencies. In addition, the authors also suggested highly to include “Bitcoin Futures” as a derivative which could discover more significant information regarding the influencing-mechanism of the connection among cryptocurrencies and stock indices in the future.

Furthermore, Omane-Adjepong et al. (2021) noted out a comprehensive future research question that suggested to find out the answer of whether the internal and/or external factors really influence the herding behaviour between cryptocurrency and emerging markets, or not. This can allow to understand the determinants that are important for consideration.

Zhang et al. (2021) advised in their future research question to assess the risk spillover between Bitcoin and disaggregated stock indexes which could provide significant findings for the investors later. However, before performing investigation on this future research question, the author highly notified to utilize Ethereum and Ripple combining with Bitcoin to better reflect the global cryptocurrency markets.

The authors suggest that all types of cryptocurrencies should be considered to explore their impact on stock market. Most of the scholars used Bitcoin in their study.

Finally, López-Cabarcos et al. (2021) have also provided an interesting future research question recommending in-depth analysis of Bitcoin behaviour. However, the study curiously advised to conduct analysis to ensure the possibility of a bidirectional connection between Bitcoin and investor sentiment. Furthermore, they also suggested to broaden the analysis by investigating the correlation between Bitcoin and other financial factors such as, currencies & indices which could convey significant ideas for future investors.

## 7 Conclusion

By using bibliometric citation analysis and content analysis, this is the first research to look at the relationship between bitcoin and the stock market. We studied a total of 151 Scopus publications published between 2008 and November 2021. We conducted the bibliometric study using the VOSviewer software. Additionally, we performed the following analyses in our systematic review: (a) identification of important features of the literature, (b) network analysis of co-authorship, (c) bibliographic coupling, (d) co-occurrence of keywords, and (e) content analysis.

This research makes contribution to the existing body of knowledge on the link between the cryptocurrency market and the stock market. To begin, we explore the evolution of cryptocurrencies, which enables us to have a better understanding of its history. Second, we explored the prominent authors, countries, journals, and institutions related to the subject. Third, we demonstrate co-authorship network analysis, bibliographic coupling, and keyword co-occurrence analysis, which all contribute to our understanding about the network. Fourth, we identify four major research areas and highlights the most important findings from each. Lastly, we have also presented seven research questions that can be addressed in the future.

The possible limitations of the study, we covered a large range of literature up to November 2021, but recent articles should be covered. Our recommendation is to revisit this procedure in a few years. Additionally, we employed just one bibliometric citation database (Scopus). Analyzing with other databases is necessary to gather more evidence about the relationship between cryptocurrency and the stock market. In this respect, we propose undertaking an investigation of the bibliometric citations between cryptocurrency and the stock market using additional databases, such as Google Scholar, Web of Science (WoS), and Dimensions, if the software is available.

## Declarations

### Author contribution statement

All authors listed have significantly contributed to the development and the writing of this article.

### Funding statement

This research did not receive any specific grant from funding agencies in the public, commercial, or not-for-profit sectors.

### Data availability statement

Data will be made available on request.

### Declaration of interest's statement

The authors declare no conflict of interest.

### Additional information

No additional information is available for this paper.

## Footnotes

1 All Cryptocurrencies - Investing.com.

2 https://www.msci.com/our-solutions/indexes/emerging-markets.

3 https://ggu.libguides.com/c.php?g=106866&amp;p=693916.
